# The Calcitonin Receptor Gene Is a Candidate for Regulation of Susceptibility to *Herpes simplex* Type 1 Neuronal Infection Leading to Encephalitis in Rat

**DOI:** 10.1371/journal.ppat.1002753

**Published:** 2012-06-28

**Authors:** Nada Abdelmagid, Biborka Bereczky-Veress, André Ortlieb Guerreiro-Cacais, Petra Bergman, Katarina M. Luhr, Tomas Bergström, Birgit Sköldenberg, Fredrik Piehl, Tomas Olsson, Margarita Diez

**Affiliations:** 1 Department of Clinical Neuroscience, Neuroimmunology Unit, Karolinska Institutet, Karolinska University Hospital, Stockholm, Sweden; 2 Department of Medicine, Infectious Disease Unit, Karolinska Institutet, Karolinska University Hospital, Stockholm, Sweden; 3 Department of Neuroscience, Neurodegenerative Diseases Unit, Karolinska Institutet, Stockholm, Sweden; 4 Department of Clinical Virology, Göteborgs Universitet, Göteborg, Sweden; Northwestern University, United States of America

## Abstract

*Herpes simplex* encephalitis (HSE) is a fatal infection of the central nervous system (CNS) predominantly caused by *Herpes simplex* virus type 1. Factors regulating the susceptibility to HSE are still largely unknown. To identify host gene(s) regulating HSE susceptibility we performed a genome-wide linkage scan in an intercross between the susceptible DA and the resistant PVG rat. We found one major quantitative trait locus (QTL), *Hse1*, on rat chromosome 4 (confidence interval 24.3–31 Mb; LOD score 29.5) governing disease susceptibility. Fine mapping of *Hse1* using recombinants, haplotype mapping and sequencing, as well as expression analysis of all genes in the interval identified the calcitonin receptor gene (*Calcr*) as the main candidate, which also is supported by functional studies. Thus, using unbiased genetic approach variability in *Calcr* was identified as potentially critical for infection and viral spread to the CNS and subsequent HSE development.

## Author Summary

*Herpes simplex* encephalitis (HSE) is a rare, but severe infection of the central nervous system (CNS) caused by *Herpes simplex* virus type 1. We have previously characterized a model for HSE in the inbred DA rat which resembles human HSE. Interestingly the inbred PVG rat is completely resistant to the disease and displays reduced or no uptake of viral particles into the peripheral and central nerve compartments respectively. To identify the gene(s) regulating HSE pathogenesis, we crossed the susceptible DA and the resistant PVG.A rats for two generations and infected 239 rats of the F2 (DAxPVG.A) cohort with HSV-1. A genome-wide linkage scan demonstrated one strong quantitative trait locus (QTL), *Hse1*, on rat chromosome 4 regulating disease susceptibility. Fine mapping, haplotype mapping, sequencing and expression analysis of the genes in the *Hse1* interval collectively support the underlying genetic variation to be located in, or adjacent to the calcitonin receptor gene (*Calcr*). Further support for a role of CalcR in regulating HSV-1 replication and propagation is provided by strain-dependent differences in the calcitonin receptor protein tissue localization and in functional studies. Using an unbiased genetic mapping approach this study identifies *Calcr* as a candidate for regulating susceptibility to HSE.

## Introduction

*Herpes simplex* type 1 virus (HSV-1) is a member of the *Herpesviridae* family (*Alphaherpesvirinae* subfamily) that infects a large fraction of humans resulting in transient cold sores or non-symptomatic infection that persists lifelong in the sensory ganglia. Recurrent herpetic disease results from reactivation of HSV-1 in the sensory ganglia subsequently leading to axonal transport of the virus to the periphery where it causes skin lesions, cold sores, often located around the mouth. However, HSV-1 can also cause a much more severe condition, *Herpes simplex* encephalitis (HSE), an acute inflammatory condition of the brain. Even though *Herpes simplex* is a neurotropic virus, HSE occurs in only 2–3 previously healthy individuals/million/year in all age groups [1]. In more than ninety percent of the cases, HSE is caused by HSV type 1 and in the remaining by HSV type 2 [2]. HSE is characterized by acute onset of focal infection, inflammation and necrosis, mostly starting unilaterally in the fronto-medio-basal temporal lobe. The disease has a tendency to relapse or to have a progressive course [3]. The mortality is high and there is significant morbidity among the survivors.

Host factors contributing to susceptibility or resistance to HSE are still largely unknown.

Genetic analysis is one approach to identify these factors. Polymorphisms in the UNC-93B and TLR3 genes were shown to regulate susceptibility to HSE in small human pedigrees, in which the production of IFN-α/β and -λ dependent on UNC-93B protein expression controls HSV-1 by TLR3-dependent and/or TLR-independent pathways [4] [5]. In addition, recently, autosomal dominant and recessive deficiencies in TRIF, an adaptor molecule involved in downstream signaling of TLRs, have been reported in a few children with HSE [6]. However, in an experimental mouse model for HSE, a natural killer (NK) complex-linked locus, *Rhs1* (resistance to Herpes simplex virus 1), on chromosome 6 has been identified to control resistance to acute and latent HSV-1 infections resulting in HSE [7]. In 2003 Lundberg and colleagues identified in another mouse model of corneal HSV-1 infection an additional locus on chromosome 6, *Hrl* (Herpes resistance locus) influencing survival after HSV-1 infection in C57BL/6J mice and the HSE development in 129S6SvEv/Tac mice [8]. Up to date, no HSE susceptibility genes have been identified by positional cloning in mice. Several mouse knock-out studies have shown the complex immune control of HSE, with excessive infiltration of leukocytes leading to the release of cytokines into the CNS suggested to be a major determinant of brain damage after infection, in turn regulating outcome [9].

The aim of the present study was to identify additional host factors determining HSE receptiveness by genetic dissection of the previously characterized discordant HSE susceptibility pattern in the inbred Dark Agouti (DA) and Piebald Virol Glaxo (PVG) rat strains [10]. This *in vivo* model for HSE in DA rats resembles in some aspects the viral spread seen in human HSE, where the virus starts spreading from the whiskers area of the rats (the labio-facial area in humans), through the trigeminal nerve to the ipsilateral side of the brain stem dispersing both to the contralateral side and towards the thalamus, causing immune activation and lethal encephalitis 5 days post-infection (dpi) [10]. Interestingly, in our previous study, PVG rats were found to be completely resistant to development of clinical symptoms and did not show evidence of penetration of virus into the brain. Although both strains showed similar virus presence in the whiskers area [10], as well as in the immediate proximity of nerve endings and small nerve fascicles [11], spread of HSV-1 to the brain was only seen in DA rats. The major histocompatibility complex (MHC) of DA or PVG did not regulate the resistance to HSE, since also MHC congenic PVG.A (RT1.AV1) rats carrying the same MHC haplotype as the DA were protected [11]. Similar results have been shown previously in studies with inbred and congenic mice where genes within the H-2 (major histocompatibility complex) did not influence resistance or susceptibility to HSV-1 infection [12] [13].

To identify gene loci critical for the strain dependent difference in HSE susceptibility, we performed a genome-wide linkage study in a large F2 (DAxPVG.A) cohort in which all HSV-1 infected F2 rats were phenotyped for disease symptoms. The study identified *Hse1* on chromosome 4 as the single strong quantitative trait locus (QTL). To find the critical gene variant within *Hse1* we performed further analyses using recombinants, haplotype mapping and gene sequencing, as well as expression analysis, visualization of the tissue localization and receptor modulation experiments.

## Results

### DAxPVG.A F2 intercross population

HSV-1 was inoculated into the right whiskers' pad of 45 days old DA rats. However, while DA rats developed a severe, lethal HSE with nearly 100% incidence at 5 days post infection (dpi), both PVG and the PVG.A rats remained completely asymptomatic even after several weeks post infection [10]. To identify gene regions causing differences in HSE susceptibility, we crossed DA with PVG.A rats for two generations to produce an F2 population. All rats of the F1 generation were resistant to the disease. In a cohort of 239 F2 (DAxPVG.A) rats infected with HSV-1 the total incidence of HSE was 15%. The remaining rats did not show signs/symptoms of disease or were only affected by minimal weight loss around 5 dpi. Stratification for gender showed 20% HSE incidence in F2 males, with mean onset of disease at 6 dpi and 10% HSE incidence in F2 females, with a slightly delayed onset of disease at 8 dpi. The studied phenotypes are summarized in Table 1.

**Table 1:** HSE incidence and body weight change in F2 (DAxPVG.A) population after HSV-1 infection.

	Females		Males	
	Diseased	Not Diseased	Diseased	Not Diseased
No. individuals	12	108	24	95
Incidence (%)	10	90	20	80
Onset (dpi)	8	−	6	−
BW change d0–d5 (%)	±0	+11	±0	+15

Incidence (diseased = clinical HSE symptoms including loss of coordination/balance, paralysis, 20% weight loss or death before d10; Not diseased = no disease symptoms).

Onset – first day of two consecutive days of weight loss or death.

BW – body weight change between day 0 and day 5.

### Quantitative trait loci (QTLs) regulating HSE incidence

**Figure 1:**
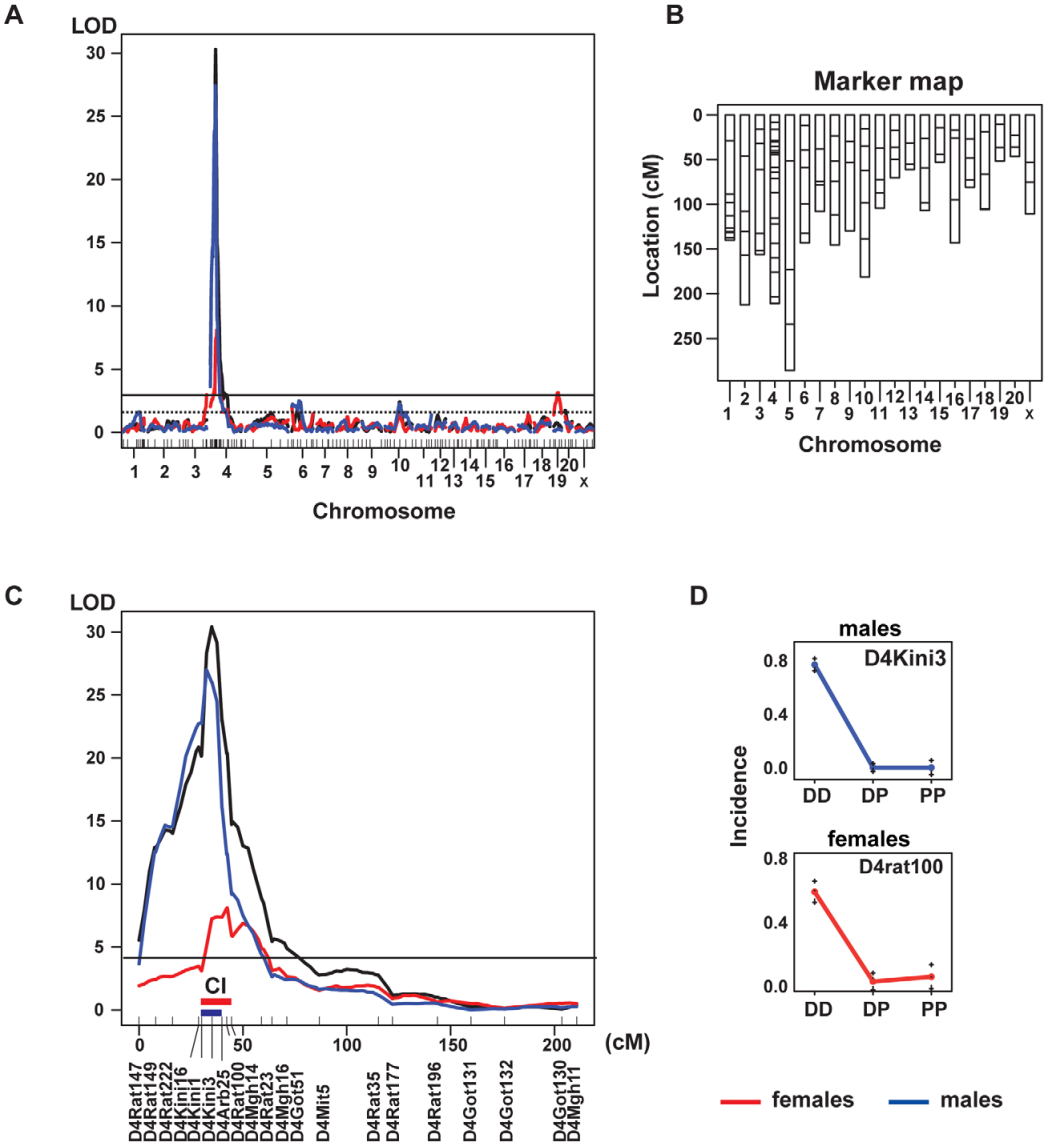
Genome-wide linkage scan of HSE incidence revealed a QTL on chromosome 4 regulating susceptibility. (A) Genome-wide linkage quantitative trait loci (QTL) map of HSE incidence in F2 (DAxPVG.A). Linkage analysis revealed a main QTL on chromosome 4 and additional significant QTLs on chromosome 3 and 10 (black curve); linkage in males (blue curve) and in females (red curve). The horizontal line: significant LOD score genome-wide threshold at *P*≤0.05. Lower dotted horizontal line: suggestive LOD score genome-wide threshold at *P*≤0.63. (B) A genetic map showing the distribution of the 127 microsatellite markers used for linkage analysis of the F2 cohort. (C) HSE main regulating QTL on rat chromosome 4, (*Hse1*) (black curve); linkage in males (blue curve) and in females (red curve). The horizontal line: significant LOD score genome-wide threshold at *P*≤0.05. The 20 microsatellite markers used for chromosome 4 linkage analysis are shown in the *x* axis and the distance between them reflects the recombination fraction in our experimental population rather than physical location. The D4Kini microsatellite makers were designed by our group. The peak marker is D4Kini3 (27.8 Mb). Thick blue horizontal bar above the *x* axis indicate the confidence interval (CI) of *Hse1* in males, whereas the thick red horizontal bar above the *x* axis indicate the CI in females. (D) The effect plots indicate that HSE incidence was driven by DA alleles in both males and females. DD = DA homozygous; DP = DA/PVG.A heterozygous; PP = PVG.A homozygous.

**Figure 2:**
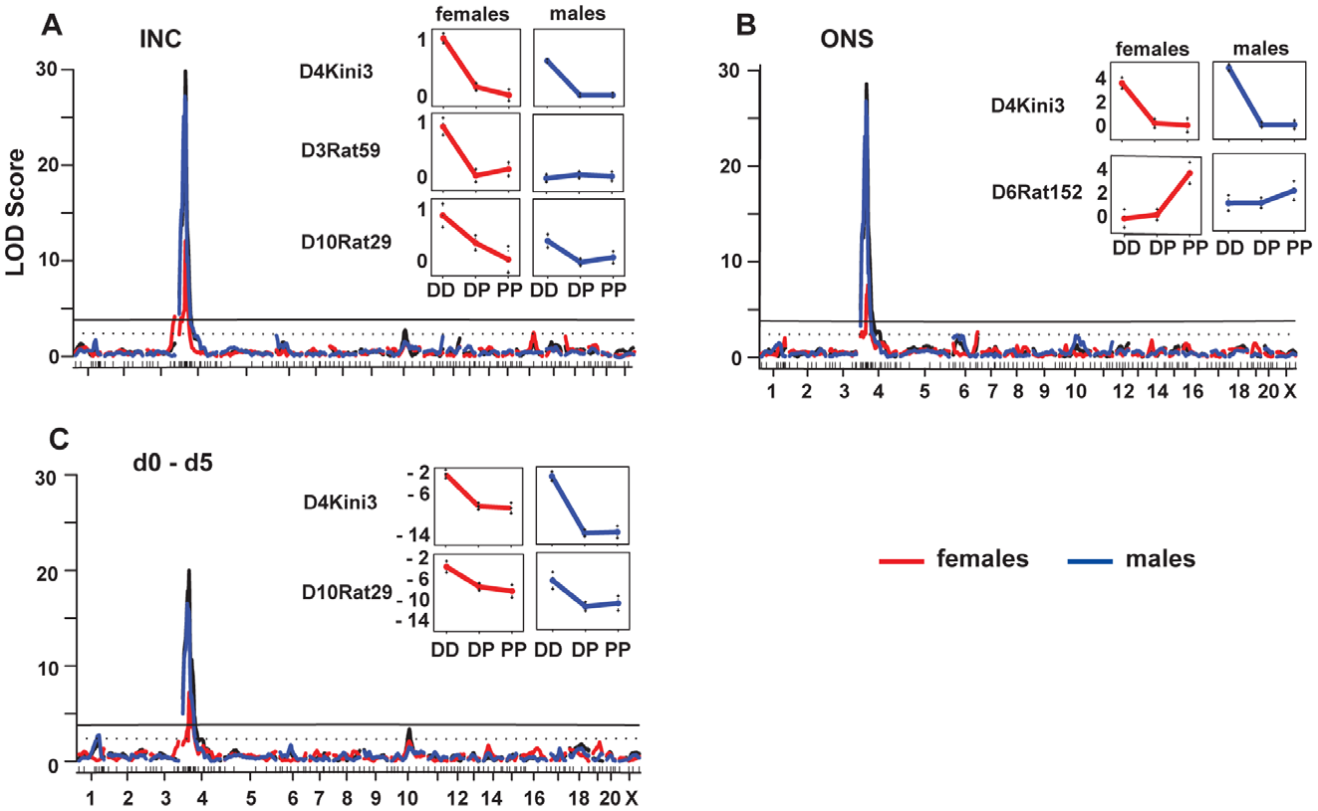
Whole genome scan and linkage analysis of the different HSE phenotypes tested in F2 (DAxPVG.A). (A–C) Genome-wide linkage QTL maps of HSE phenotypes (black curve); linkage in males (blue curve) and in females (red curve). Upper horizontal line: significant LOD score genome-wide threshold at *P*≤0.05. Lower dotted horizontal line: suggestive LOD score genome-wide threshold at *P*≤0.63. To the right of each graph are the effect plots for each QTL indicating the alleles in both males and females, which drive the HSE phenotypes. DD = DA homozygous; DP = DA/PVG.A heterozygous; PP = PVG.A homozygous. A – incidence of HSE; B – onset of disease; C – weight loss d0–d5.

A genome-wide linkage scan was performed using 127 microsatellite markers on 180 F2 (DAxPVG.A) rats to determine the genomic region(s) influencing susceptibility to HSE. We found a very strong linkage to a region on chromosome 4, designated *Hse1* which regulated the incidence of encephalitis with a logarithm of odds (LOD) score of 29.5 at the D4Kini3 marker located at 27.8 Mb (Figure 1, Table 2). The confidence interval (CI) of *Hse1* is between D4Kini1 and D4Arb25 (24.3–31.1 Mb) (Table 2). The linkage strength was reduced when analyzing females only, with a LOD score of 12.1. In males the LOD score was 26.4 (Figure 1C). In the entire cohort, 46 rats out of 55 being homozygous for DA alleles at the peak marker developed HSE (84% incidence). In contrast, only 4 rats out of 91 homozygous for PVG alleles in *Hse1* developed HSE (4% incidence), while none of the 34 heterozygous rats developed HSE (Figure 1D). Another QTL on chromosome 3 (*Hse2*) was found to be significant for HSE incidence in females (Figure 2A, Table 2).

**Table 2:** QTLs regulating HSE phenotypes in F2 (DAxPVG.A).

QTL	Chr	Significance level	Phenotype	LOD	Peak marker	Position cM	Position Mb	Confidence interval	Position Mb
*Hse1*	4	significant	Incidence^f^	12.1	D4Kini3	35.1	27.8	D4Kini1 – D4Arb25	24.3–31.1
			Incidence^m^	26.4	D4Kini3	35.1	27.8	D4Kini1 – D4Arb25	24.3–31.1
			d0–d5^f^	7.3	D4Kini3	35.1	27.8	D4Kini1 – D4Arb25	24.3–31.1
			d0–d5^m^	16.0	D4Kini1	30.1	24.3	D4Rat222 – D4Arb25	19–31.1
			Onset^f^	7.3	D4Kini3	35.1	27.8	D4Kini1 – D4Mgh16	24.3–61.6
			Onset^m^	25.3	D4Kini3	35.1	27.8	D4Kini1 – D4Arb25	24.3–31.1
*Hse2*	3	significant	Incidence^f^	4.1	D3Rat59	156.1	163.4	D3Rat6 – end	147.9 – end
*Hse4*	6	suggestive	Onset^f^	2.6	D6Rat152	143.2	142.4	D6Rat100 – end	100.7 – end
*Hse5*	10	suggestive	d0–d5^f^	2.2	D10Rat29	98.4	68.8	D10Rat78–D10Rat219	43.3–81.9

Values represent LOD scores for main effect QTLs. Significance thresholds for each phenotype, was generated with 1000 permutations, LOD scores reaching the 95% threshold value were considered significant and LOD scores reaching the 63% threshold value were considered as suggestive. The marker closest to the position that showed the maximum LOD for each trait was denoted the peak marker. D4Kini1 and 3 represents the names of the designed microsatellite markers; ^“f”^ the phenotype calculated in females. ^“m”^ the phenotype calculated in males.

### QTLs regulating other HSE phenotypes

In addition to HSE incidence, the onset of disease and the body weight change were recorded as phenotypes. Body weight change was determined as the difference between the weight at the day of infection and weight at 5 dpi (d0–d5) (Table 1). The locus *Hse1* was the main regulator of the onset of disease, which also correlated to body weight change between d0–d5 (Figure 2B and 2C). *Hse4* and *Hse5* located on chromosomes 6 and 10, showed suggestive linkage to disease onset and weight change between d0–d5 in females, respectively (Figure 2B and 2C). Notably, for all these QTLs linkage was stronger in males and the effect plots showed disease susceptibility associated with DA alleles. These QTLs are summarized in Table 2.

A major host genetic contribution to HSE susceptibility was provided by the *Hse1* locus, contributing with 51% in females and 50% in males to the variance of incidence and further contributing with 54% in females and 64% in males to the variance of onset. Additional interactive QTLs specific for each sex were identified in a number of chromosomes collectively contributing to more than 90% of the variance in most phenotypes in females and less in males, indicating a more complex regulation of HSE in females (Table S1).

### *Hse1* regulated HSE incidence in congenic lines

**Figure 3:**
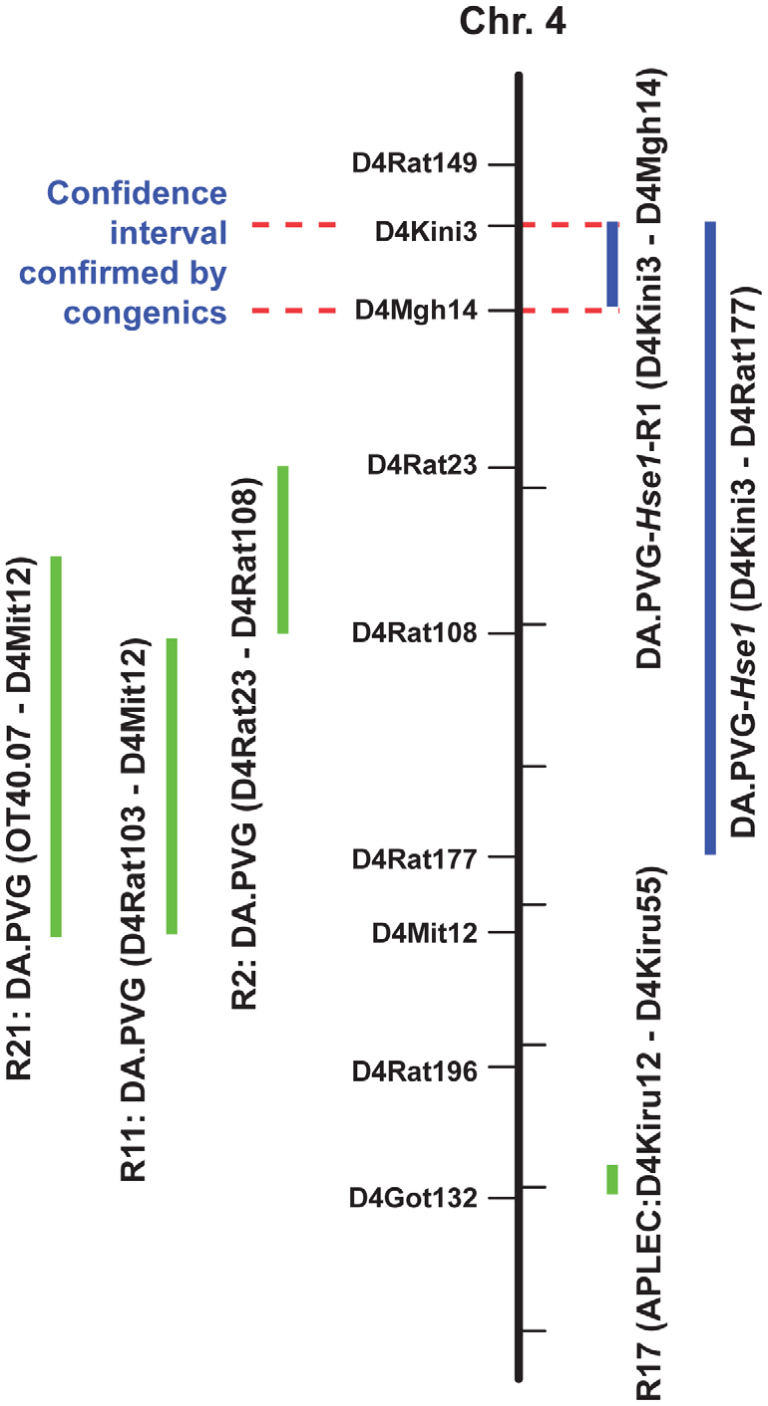
HSV-1 infected congenic lines confirm HSE regulation by *Hse1*. A 6.8 Mb region on rat chromosome 4 contains a QTL regulating HSE susceptibility. The black vertical bar represents rat chromosome 4 with a number of microsatellite markers used. The blue vertical bars represent the congenic lines we developed DA.PVG-*Hse1*, with a PVG fragment (D4Kini3 – D4Rat177) transferred to DA background and DA.PVG-*Hse1*-R1, with a PVG fragment (D4Kini3 – D4Mgh14) transferred to DA background, containing the region of interest *Hse1*. All infected rats from these strains were completely protected from HSE development. The green vertical bars represent the chromosome 4 congenic lines R2, R11, R21 (*Eae*) and R17 (APLEC) with PVG fragments on DA background available in our laboratory, which were used to test for HSE incidence. These strains developed HSE as DA rats. The non-overlapping fragment between DA.PVG-*Hse1*, R2, R11 and R21 congenic lines delineates the disease regulatory effect of *Hse1* to a region between D4Kini3 – D4Rat23, which is excluded by the horizontal red dotted lines.

To obtain direct experimental proof that gene(s) in the region indeed regulate HSE susceptibility, and for further genetic dissection we bred a set of congenic lines. These lines included DA.PVGc4-*Hse1* and DA.PVGc4-*Hse1*-R1, which carries the PVG fragment between the microsatellite markers D4Kini3 – D4Rat177 and D4Kini3 – D4Mgh14, respectively, transferred onto DA background. In order to test if PVG alleles in these fragments confer resistance to HSE, 5 homozygous rats from each congenic strain were infected. None of the rats developed symptoms, demonstrating the protective role of the PVG alleles in the *Hse1* region (Figure 3). For both mapping and control purposes, we furthermore tested HSE susceptibility in a set of congenic lines with different chromosome 4 congenic PVG fragments on the DA background; R2:DA.PVG (D4Rat23–D4Rat108), R11:DA.PVG (D4Rat103 – D4Mit12), R21:DA.PVG (OT40.07 – D4Mit12) covering the experimental autoimmune encephalomyelitis (EAE) QTLs Eae24–Eae27 [14] and R17 (D4Kiru12–D4Kiru55) covering the APLEC genes region that are associated with arthritis and autoimmunity in rats and humans [15] (Figure 3). All of these lines are homozygous for DA alleles in *Hse1*. All four lines developed disease with a clinical phenotype inseparable from that of DA rats, providing further support for the disease regulatory effect to be located within *Hse1*. Notably, the non-overlapping fragment between on the one hand DA.PVGc4-*Hse1* and on the other hand the R2, R11 and R21 congenic lines delineates the disease regulatory effect of *Hse1* to a region between D4Kini3 – D4Mgh14 (Figure 3).

### Fine mapping of the *Hse1* disease regulatory effect

**Figure 4:**
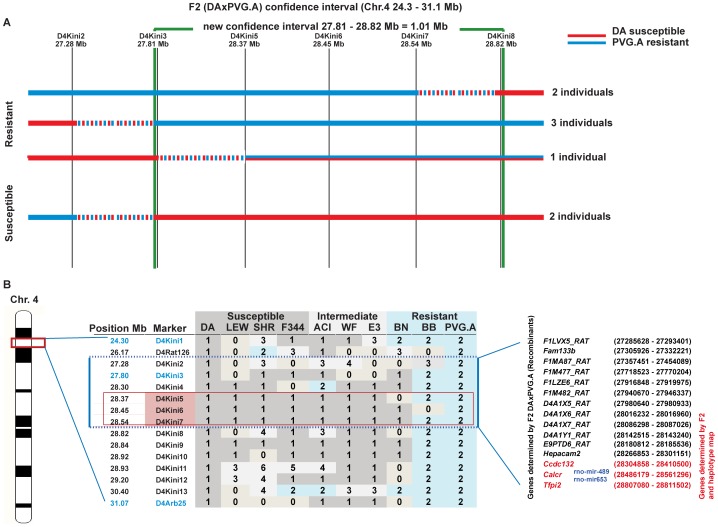
*Hse1* confidence interval narrowing by using more F2 (DAxPVG.A) and Haplotype mapping other inbred strains. (A) The use of newly designed microsatellite markers in *Hse1* F2 (DAxPVG.A) and the few other F2 rats identified to recombine in the *Hse1* region. Red horizontal bars represent DA alleles and the blue bars PVG.A alleles. Intermittent red/blue dashed bars represent the interval of recombination between two markers. The green vertical bars delineate the new confidence interval (D4Kini3–D4Kini8). (B) Haplotype map presents the genotype in *Hse1* region of the different inbred rat strains tested for HSE development. DA genotype = 1; PVG.A genotype = 2. Other numbers indicate different genotypes. The left and right blue vertical bars together with the 2 blue horizontal dotted lines represent the CI defined by F2 recombinant rats (D4Kini2–D4Kini7). The pink panel shows the suggested CI defined by the haplotype map (D4Kini5–D4Kini7) where susceptible and intermediate strains shared the same genotype as DA. The red frame points out the region, which is most likely regulating HSE. The list of the genes within the narrowed F2 recombinants CI and their genomic position in megabases are shown to the right side of the figure, the genes defined by F2 recombinants and haplotype map are marked in red; and the miRNAs within the *Calcr* gene are shown in blue as described in the Ensembl release 66.

To fine map the regulatory effect within the large CI of *Hse1* obtained from the initial F2 linkage analysis (24.3–31.1 Mb∼6.8 Mb region), the entire initial F2 population together with additional F2 rats recombinant in the CI from a parallel intercross were genotyped with more microsatellite markers within the CI (D4Kini2, D4Kini4–D4Kini16) (Figure 4A, Table S2). By defining the recombination positions within the CI region in recombining F2 rats, we were able to narrow the CI into a smaller region where resistant and susceptible rats have different genotypes. The fine mapping in F2 rats narrowed the CI of *Hse1* to 1.01 Mb (CI D4Kini3–D4Kini8; 27.81–28.82 Mb) (Figure 4A).

To further define the CI and the exact region governing the disease susceptibility, we determined the development of HSE symptoms in a panel of other inbred rat strains and correlated their susceptibility to the allelic variation in this region (Figure 4B). We found that the Lewis (LEW), Fisher 344 (F344) and Spontaneously Hypertensive Rat (SHR) were susceptible to HSE and developed a similar disease phenotype as DA, while the Bio Breeding type 1 diabetic rat (BB) and Brown Norway (BN) in similar to PVG were resistant. Other inbred strains developed a different disease pattern from DA, where some rats developed HSE symptoms while others displayed only mild or no symptoms of disease. These strains were considered to develop an intermediate phenotype, and included the August Copenhagen Irish (ACI), Wistar Furth (WF) and Fawn-Hooded (E3) strains (Figure 4B). Based on the haplotype map in this larger set of inbred strains, the disease regulatory effect was suggested to be located within a smaller, 0.17 Mb region (CI D4Kini5–D4Kini7; 28.37–28.54 Mb) (Figure 4B, Table S2). However, the haplotype map was based on the susceptibility to HSE and the development of clinical symptoms in each strain after infection and not the viral presence in the CNS. The haplotype map also suggests a more complex genetic regulation of susceptibility *vs.* resistance to HSE, since the resistant BN strain carries the same genotype in this region as the susceptible strains (Figure 4B). This supports the notion that different genes might be involved in regulating the clinical phenotype of HSE as well as pattern of virus spread across different strains.

In the Ensembl release (version 66; http://www.ensembl.org/) the genomic region defined by F2 recombination (CI D4Kini3–D4Kini8; 27.81–28.82 Mb) contains the genes cyclin-dependent kinase 6 (*F1MA87_RAT*), hepacam family member 2 (*Hepacam2*), calcitonin receptor (*Calcr*) and tissue factor protein inhibitor 2 (*Tfpi2*), as well as 11 additional poorly annotated genes. The region also includes 3 microRNAs; novel miRNA (ENSRNOG00000043729) and known miRNAs (rno-miR-489 and rno-miR653) which are located within the *Calcr* gene. However, the region narrowed by the haplotype map (CI D4Kini5–D4Kini7; 28.37–28.54 Mb) includes only the three genes *Ccdc132*, *Calcr* and *Tfpi2* (Figure 4B).

### *Hse1* SNP variations and high mRNA expression of *Calcr* in PVG

**Figure 5:**
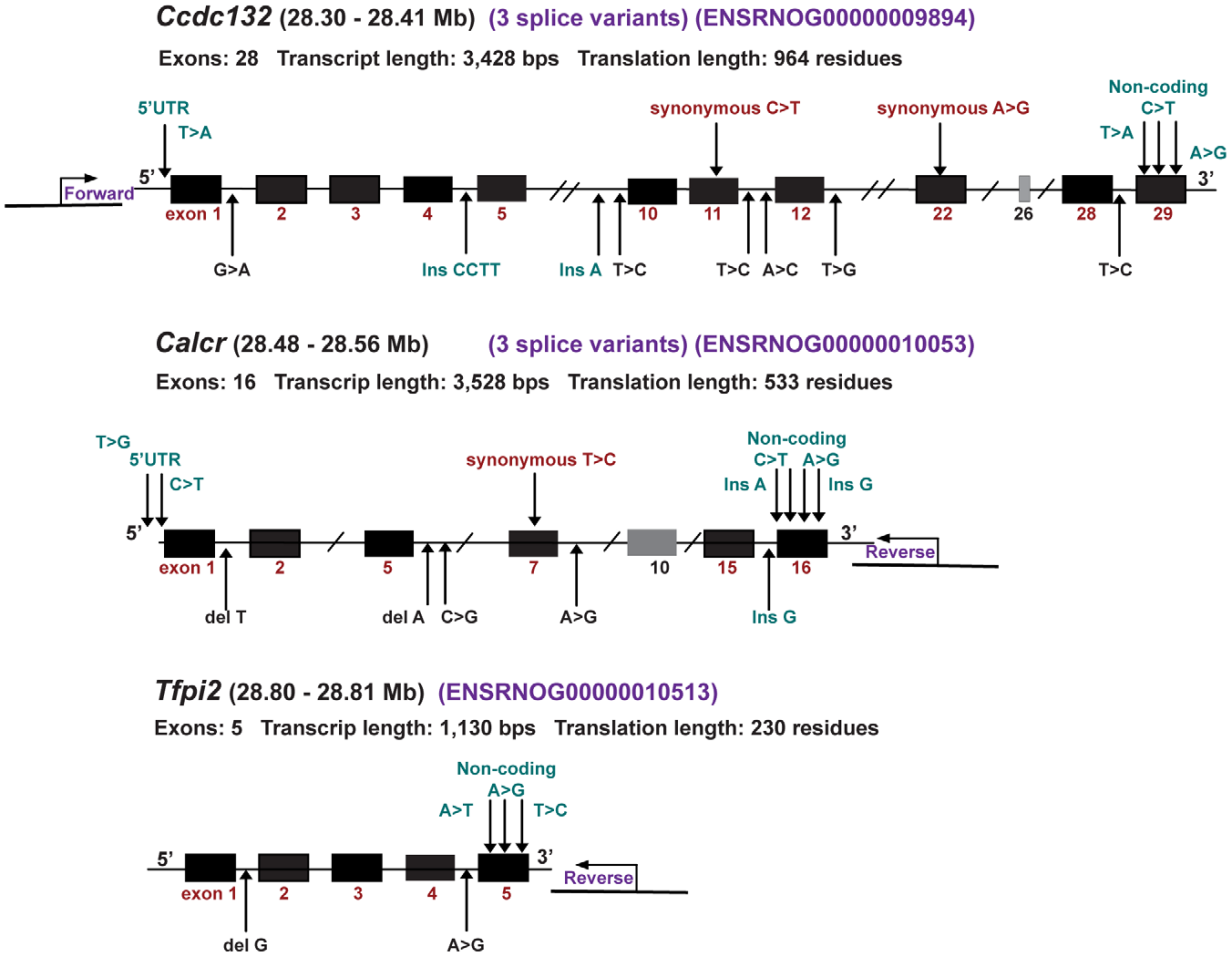
Sequences of candidate genes. Schematic illustration of genomic DNA sequences of the *Ccdc132*, *Calcr* and *Tfpi2* genes showing some of the SNP variations in PVG.A rats. Black boxes represent the different exons in each gene; the arrows below represent SNPs in intronic regions; arrows above represent SNPs in exons and 5′ UTR regions.

All genes within the recombinant CI region were sequenced from genomic DNA of DA and PVG.A rats. Comparison of the DA and PVG sequences to the BN reference sequence revealed about 1470 SNP variations in the PVG sequence in *Hse1*. The majority of the sequence polymorphisms were detected within the *Ccdc132*, *Calcr* and *Tfpi2* genes. These variations were mostly insertions/deletions in the intronic regions, SNPs in 5′ untranslated regions and intergenic regions. Only 4 synonymous SNPs in exons were detected (Figure 5). In addition, no SNPs were found in pre-miRNA sequences of miR-489 and miR-653 when comparing the DA and PVG with the reference genome (data not shown).

**Figure 6:**
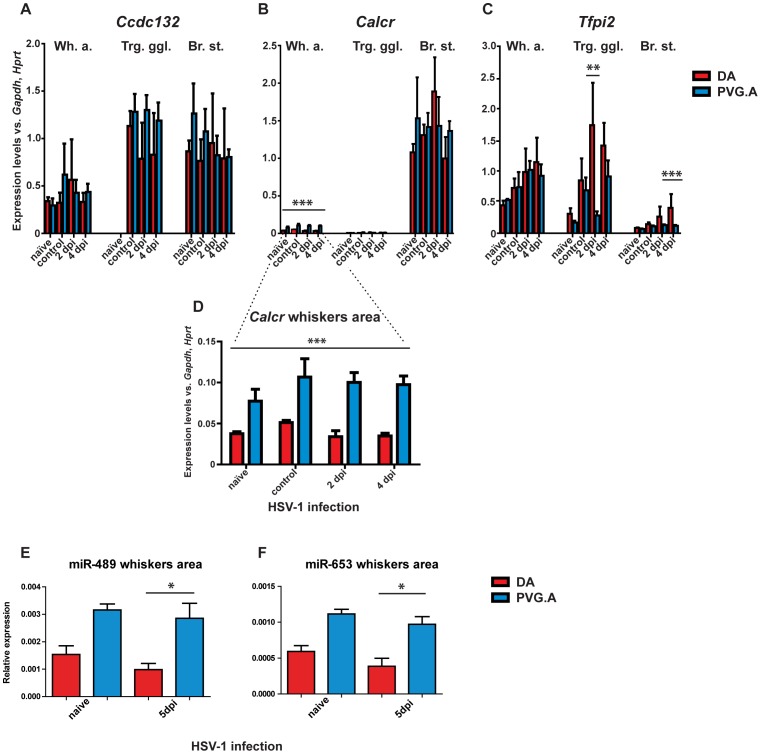
mRNA expression of candidate genes and miRNA expression. The mRNA expression analysis of the candidate genes (A) *Ccdc132*, (B) *Calcr* and (C) *Tfpi2* genes in the whiskers area (Wh. a.), trigeminal ganglia (Trg. ggl.) and brain stem (Br. St.) in naïve rats (n = 3), vehicle-injected controls (n = 5) and HSV-1 infected rats after 2 (n = 5) and 4 (n = 5)dpi of DA (red) and PVG.A (blue) rats. (D) The mRNA expression analysis of the *Calcr* showed significantly higher values in the resistant PVG.A strain in the whiskers area of naïve, vehicle-injected controls and HSV-1 infected rats at all time points. The dotted lines coming from (B) to show that it is the same figure. Significance was determined using two-way ANOVA, with Bonferroni post-hoc test. **P<0.01; ***P<0.001. The miRNA expression of (E) miR-489 and (F) miR-653 in the whiskers area of naïve DA (n = 4), DA 5 dpi (n = 5) (red), naïve PVG (n = 5) and PVG 5 dpi (n = 5) (blue). The expression of miR-489 and miR-653 was significantly higher in PVG.A rats in the whiskers area compared to DA rats at 5 dpi. Significance was determined using one-way ANOVA, with Kruskal-Wallis test. *P<0.05.

In order to study if these polymorphisms might affect translational stability, splicing or transcriptional control, we determined the mRNA expression pattern for *Ccdc132*, *Calcr* and *Tfpi2* in the whiskers area, the trigeminal ganglia and the brain stem using qRT-PCR (Figure 6A–D). The most conspicuous finding was that PVG.A rats displayed higher *Calcr* expression in the whiskers area both in all controls as well as after HSV-1 infection compared to DA (Figure 6B and 6D). Little or no expression of *Calcr* was detected in the trigeminal ganglia in both strains, indicating that the influence of *Calcr* is located in the periphery. *Calcr* has three splice variants; the primer used in Figure 6 covered exons common for all the variants. No differences in expression between the strains were detected when measuring the splice form of *Calcr.1b*, however it was expressed more in the brain, with no or low expression in the trigeminal ganglia and the whiskers area, respectively (data not shown). The expression of *Tfpi2* was significantly higher only after infection in the trigeminal ganglia (2 dpi) and the brain stem (4 dpi) in DA rats compared to PVG rats. This could partly be explained by the presence of the virus in these compartments, since no difference was detected in the periphery. Primers used for qRT-PCR are listed in Table S3.

As for the two miRNAs encoded in one of the introns of *Calcr*, mir-489 and mir-653, the expression of both the mature and the mature* miRNA was measured in the whiskers area of naïve and 5 dpi rats using qRT-PCR. The expression of both the mature mir-489 and mir-653 was significantly higher in the PVG rats at 5 dpi compared to the DA rats (Figure 6E and 6F). As expected, the expression of both mature* miRNAs was very low, showing a similar expression pattern to the mature miRNAs, with significantly higher expression of mir-653* in PVG rats at 5 dpi as the only detected strain difference (data not shown). Notably, the expression of the two miRNAs followed the mRNA expression pattern of *Calcr* in the whiskers area, suggesting that they are not expressed independently but rather as a result of *Calcr* expression.

### *Calcr* is expressed in the peri- and endoneurium of DA rats after HSV-1 infection

To visualize differences in viral spread and tissue immune response compared to the tissue-location of CalcR, the whiskers area was dissected and subsequently processed for staining using immunohistochemistry. Tissues from DA and PVG.A strains were dissected from naïve, Hank's solution-injected controls and infected rats after 5 dpi. Results are summarized in Table 3.

**Table 3:** Summary of immunohistochemistry staining of the whiskers area of DA and PVG.A rats infected with HSV-1, at 5 dpi.

Marker	Phenotype	DA Naïve	DA 5 dpi	PVG.A Naïve	PVG.A 5 dpi	Result staining
HSV-1	Virus staining	−	++	−	+	In endoneurium, perineurium (DA) and axons (DA)
CalcR	Overall staining	+++	+++	++++	++++	In connective tissue, perineurial cells
CalcR	Clusters in nerves	−	+	−	+++	Clusters associated with HSV-1
CalcR	Endoneurium	−/++	−/++	−	−	In the nerve
CalcR+peptide	Absorption 10^−6^ M	−	−	−	−	In connective tissue, nerves (unspecific: muscle fibers)
NKR	Natural killer cells	+	+++++	+	++	In connective tissue
CD8	T-cells	−	++++	−	+++	In connective tissue, HSV-1 negative,
Iba1	Macrophages, dendritic cells	+	++++	+	++++	In cells in the close proximity to the injection site
MHC class I		−/+	++	−/+	++	In infiltrating cells

**Figure 7:**
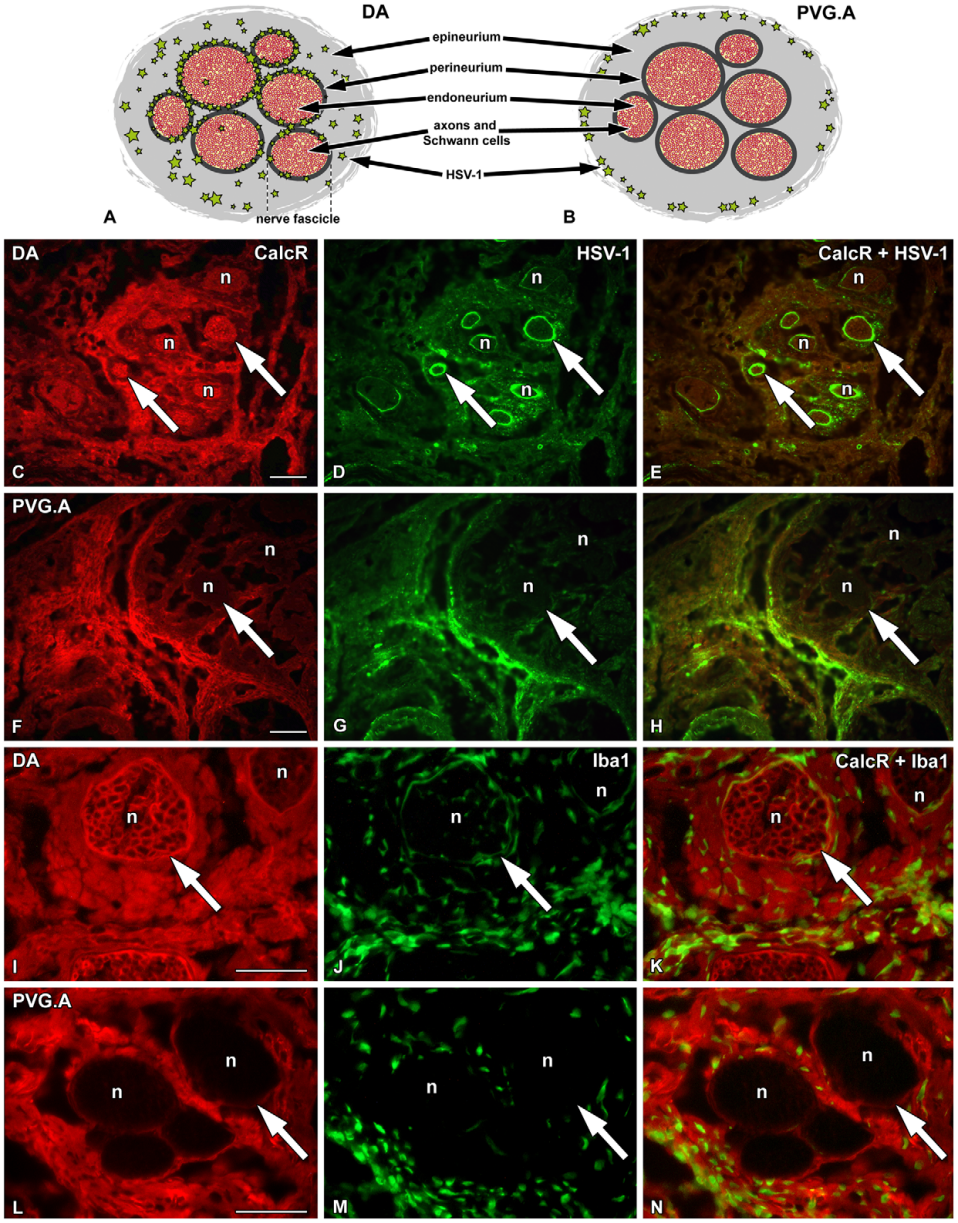
Calcitonin receptor is expressed in the peri- and endoneurium of DA rats after HSV-1 infection. Schematic drawings of the peripheral nerve structure in the whiskers area and the localization of the HSV-1 staining in the two rat strains (A and B) and immunofluorescence micrographs illustrating sections of the whiskers area at 4 dpi. The DA (C–E and I–K) and the PVG (F–H and L–N) sections were stained with CalcR marker (red; C, E, F, H, I, K, L and N), HSV marker HSV-1 (green; D, E, G and H) and macrophage marker Iba1 (green; J, K, M and N). (C and E) In DA rats CalcR was expressed in all compartments of the infected nerve fascicles, where also HSV-1 (D and E) was detected. (G and H) In PVG rats HSV-1 penetrated only to the outer part of the epineurium. (F and H) CalcR expression was mostly detected in the outer part of the epineurium. (I and K) In DA rats CalcR was expressed in a honeycomb-like pattern easily observable at a higher magnification. (J and K) Iba1^+^ macrophages penetrated into all compartments of the affected nerve fascicles in DA rats. (L and N) In PVG rats high expression of CalcR could be observed in the outer part of the epineurium. (M and N) Penetration of macrophages was also restricted to the outer part of the epineurium. Large arrows point to the nerve fascicles. n = nerve fascicle. Scale bar: 50 µm.

In a previous study [11], we have shown that HSV-1 staining was similarly distributed in the whiskers area after infection in PVG and DA rats, but subsequently it became strongly increased, mostly in the perineurium, in DA rats (Figure 7A), *i.e.* the layer of connective tissue surrounding the nerve fascicles/bundles of peripheral nerves. In contrast, in PVG.A rats replication of virus decreased in the whiskers area after 2 dpi (Figure 7B). HSV-1 staining was only found in DA rats in the trigeminal ganglia and the brain stem, while in PVG.A rats these compartments were completely free of HSV-1. In sections from the whiskers area at 5 dpi (Figure 7), virus labeling was present in the epineurium, *i.e.* the outermost layer of connective tissue surrounding several nerve fascicules/bundles, in both DA (Figure 7D and 7E) and PVG.A rats (Figure 7G and 7I). However, DA rats also displayed positive staining within the peri- and endoneurium, *i.e.* in the layer of connective tissue surrounding each nerve fascicle and nerve fiber inside the fascicles, respectively.

CalcR staining was stronger in the whiskers area of naïve and infected PVG.A rats compared to DA rats corroborating the qRT-PCR findings (Table 3). Interestingly, the tissue distribution of CalcR staining differed between the infected DA and PVG.A rats. In DA rats (Figure 7C, 7E, 7I and 7K), CalcR staining was present mainly in perineurial cells and in the endoneurium, while in PVG.A (Figure 7F, 7H, 7L and 7N) rats it was expressed more in the outer part of the epineurium. Thus, the distribution pattern of CalcR staining resembles that of HSV-1 in both DA and PVG.A rats at 5 dpi.

The macrophage marker Iba1 (Figure 7J, 7K, 7M and 7N) also followed the distribution pattern of HSV-1 and CalcR in each strain. Larger numbers of natural killer (NK) cells and CD8^+^ T cells were present in the whiskers area of DA compared to PVG.A rats. Infiltrating NK cells outnumbered the CD8^+^ T cells in the DA rats at 5 dpi (Table 3).

### *In vivo* modulation of CalcR in DA rats

To investigate the influence of *Calcr* on HSV-1 entry and spread to the CNS *in vivo* in DA rats, we modulated the calcitonin receptor by injecting rat Amylin, an agonist for calcitonin receptor, or Calcitonin (8–32) (Salmon I), a potent and selective antagonist [16], into the whiskers area prior to the infection. A scrambled peptide (similar to the amino acid composition of rat Amylin, but in reverse order) was used as control to the rat Amylin. Treatment was performed in 44 days old DA rats, 18 hours before the infection with HSV-1. The rats were examined and weighed every day from the day of the injection with the modifying substances until 11 dpi, to check for signs and symptoms of disease development.

**Figure 8:**
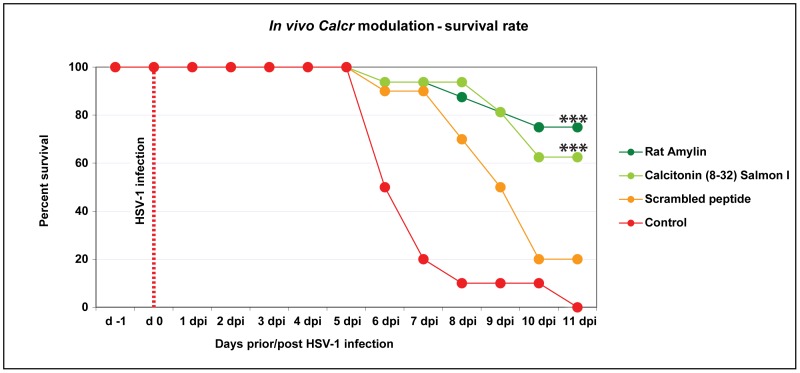
*In vivo* modulation of CalcR by rat Amylin, improved significantly the survival of DA rats. Kaplan-Mayer curves representing the survival rate of the DA rats pre-treated with CalcR agonist rat Amylin (dark green), CalcR antagonist Calcitonin (8–32) Salmon I (light green), Scrambled peptide – *i.e.* the same amino acid sequence, but in reverse order (orange line) and untreated controls only infected with HSV-1 (red). The survival rate in the groups pre-treated with rat Amylin and with Calcitonin (8–32) Salmon I had a survival rate which was significantly higher than that of the untreated controls, which all died.

Treatment with rat Amylin significantly improved the survival of the DA rats from 0 to 75% after infection compared to the control group, which was only HSV-1 infected. The survival rate in rats treated with Calcitonin (8–32) (Salmon I) group was 62%. In contrast, only 20% of rats injected with the scrambled control peptide survived until day 11 after infection (Figure 8) and all the control rats died. Collectively, these findings suggest that *in vivo* modulation of the *Calcr* in the whiskers area affects viral entry to the CNS and progression to HSE in DA rats.

### Modulation of HSV-1 infection *in vitro* by CalcR transfection

To study susceptibility mechanisms at the molecular level, we performed infections of primary neuronal cell cultures from DA and PVG rats' dorsal root ganglia. No differences in susceptibility to infection were detected in these cells *in vitro* (data not shown), indicating that *in vivo*, other features of the natural environment of the neurons contribute to the susceptibility or tolerance to infection.

**Figure 9:**
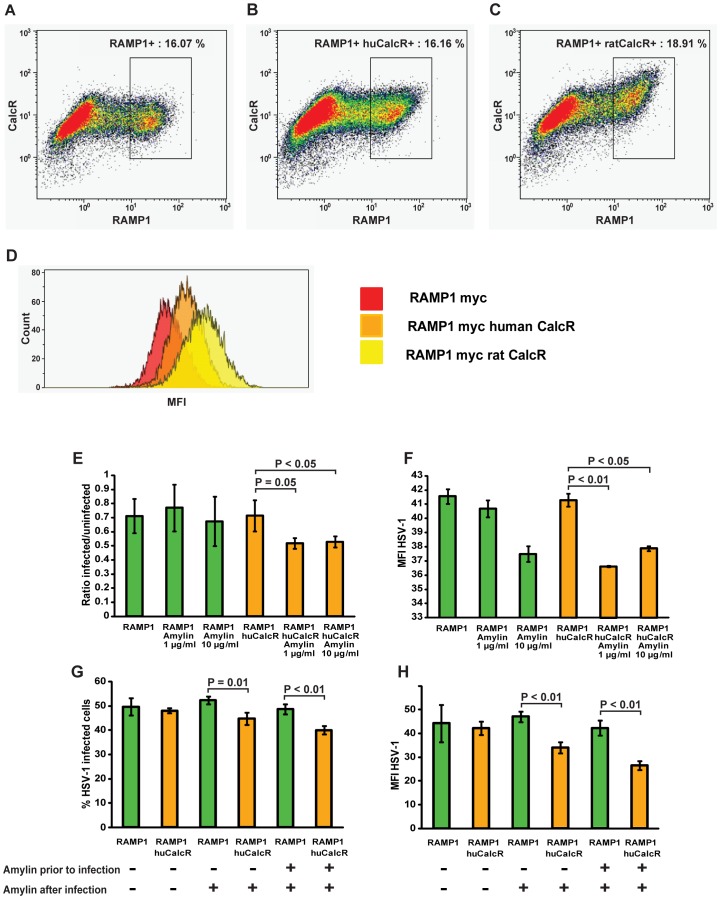
Signaling through CalcR inhibits virus replication in HEK293T cells. HEK293T cells were transfected for 24 hours with plasmids encoding for either RAMP1 alone (A), or in combination with the human CalcR (huCalcR) (B) or rat CalcR (ratCalcR) (C). (A–C) Double staining showed that CalcR was only expressed in RAMP1-positive cells. Albeit detected at a lower level than rat CalcR, huCalcR was distinctly expressed. (D) RAMP1 or RAMP1/huCalcR transfected cells were infected for 30 min with HSV-1 and virus replication was detected 24 h later with antibodies against HSV-1 structural proteins. (E) Ratio between RAMP1 or RAMP1/huCalcR infected and non-infected cells was shown for infection alone, or in the presence of 1 µg/ml or 10 µg/ml rat Amylin. (F) Analysis of the mean fluorescence intensity (MFI) of staining for HSV1 structural proteins in the infected populations. Effect of rat Amylin added prior, during and after infection as compared to incubation post-infection only (G and H). The effect on percentage (G) of RAMP1- or RAMP1/huCalcR-positive and HSV-1-positive cells, or the MFI of the infected population (H) are shown. All analyzes were done in triplicates of transfected and infected wells.

In addition to assess whether CalcR expression could modulate infectivity *in vitro*, HEK293T cells, which are semi-permissive to HSV-1 infection, were transfected with a plasmid encoding for a myc-tagged version of RAMP1, a receptor activity-modifying intracellular protein which is transported to the cell surface by CalcR and is necessary for the responsiveness of CalcR to amylin, alone or in combination with plasmids encoding for the human or the rat CalcR (Figure 9). Staining for myc-tag and CalcR showed that in co-transfection experiments only RAMP1 expressing cells also co-expressed the CalcR (Figure 9A), even though detection of the human CalcR was weaker, possibly due to lower reactivity of the antibody with the receptor form found in humans (Figure 9B). Transfected cells were then infected with HSV-1 for 24 hours and HSV-1 infection and replication were assessed by intracellular staining and flow cytometry. RAMP1 or RAMP1/Human CalcR transfected cells were analyzed (Figure 9A–9C) for the percentage of HSV-1infected cells, which indicates how permissive the cells were to the virus, as well as for the mean fluorescence intensity (MFI) of HSV-1 staining in the infected population, which reflects the level of virus replication in these cells (Figure 9D). As shown in (Figure 9E–9H), no change in the proportion of HSV-1 infected cells could be seen between RAMP1 only or RAMP1/human CalcR transfected cells, indicating that in these semi-permissive cells the expression of CalcR did not alter virus uptake and internalization. However, the addition of rat Amylin (calcitonin agonist) reduced both the number of cells infected by HSV-1 (Figure 9E) as well as the MFI of staining for structural viral components in the infected cells population (Figure 9F). The experiment was repeated with transfection of the rat CalcR yielding similar results (data not shown). To assess whether the kinetics of CalcR triggering could affect virus replication, transfected cell cultures were incubated with rat Amylin at different time points (before, during and after infection, or only after infection) and the same analysis was performed (Figure 9G and 9H). As expected from the previous experiment, rat Amylin significantly decreased the infectivity as well as the intensity of staining for virus structural components in cells that were co-transfected with RAMP1 and CalcR, but not in those transfected with the receptor modifier RAMP1 alone. In addition, the effect was slightly more pronounced in the cultures that were pretreated with rat Amylin prior to infection. Experiments involving the CalcR antagonist Calcitonin (8–32) (Salmon I) were performed in transfected HEK293T cells as described for rat Amylin but showed no modulator effect *in vitro* suggesting that the antagonist has an alternative mode of action *in vivo* (data not shown). Finally, to assess whether the CalcR could serve as a receptor or co-receptor for virus entry in a non-permissive *in vitro* system, we utilized a rat adeno-carcinoma cell line (CRL-1666) that cannot be readily infected with HSV1 and transfected and infected it in the same way as described above for HEK293T cells. Regardless of CalcR expression, these cells remained non-permissive, ruling out a role of CalcR as a receptor for virus infection *per se* (data not shown).

## Discussion

We here demonstrate a potent genetically regulated host factor critical for *Herpes simplex* virus type-1 neuro-invasion mapping to a small genome fragment on rat chromosome 4. Though very strong circumstantial evidence suggest genetic variants of the *Calcr* gene to be responsible, ultimate proof will require further experiments.

HSV-1 infects the majority of the population inducing cold sores in affected individuals. Human necropsy studies suggest that viral DNA can be isolated from nearly all post-mortem brains, implying that with time most individuals are infected. However, in younger people (between 20 to 49 years of age) HSV-1 serology suggests that only 50–60% have been infected, as reported by independent studies in different populations [17], [18], [19]. These studies also demonstrate an increasing prevalence of HSV-1 infection with age. Thus, different susceptibility patterns to establish HSV-1 infection are possible in the human population. Therefore, our findings in the rat are not necessarily in contradiction to the situation in humans. Alternatively, the differences in susceptibility through peripheral nervous system (PNS) uptake and transport of the virus, as here documented in the rat, species are not relevant for human herpes infection. A third possibility is that the molecule is not subject to genetic variation in humans, but still being important mechanistically for viral-host interactions.

On the other hand, in two to three individuals per million per year, the virus infection leads to a much more devastating condition with invasion and replication of virus in the CNS, causing focal necrotizing plaques affecting primarily the temporal and inferior frontal lobes of the brain. Although rare, HSE remains the most common cause of acute, sporadic viral encephalitis in the Western world [20]. The underlying host determinants regulating HSE susceptibility are largely unknown.

The entry of HSV-1 into the host cells is known to depend on the interaction of several glycoproteins on the surface of the enveloped virus with receptors on the surface of the host cell. These entry receptors include Herpes virus entry mediator (HVEM), nectin-1 and 3-O sulfated heparan sulfate [21]. Through these known entry receptors the virus remains latent in sensory neurons. However, still little is known about the host factors that influence reactivation and the different entry ports of HSV-1 into the CNS.

The *in vivo* model used in this study in 45 days old rats simulates the human infection in a number of aspects. It starts from the whiskers area corresponding to the labio-facial area in humans. Interestingly, DA rats at an age of over 60 days at infection were resistant to HSE disease phenotype development, while PVG rats below an age of 30 days were susceptible (unpublished observation). Age dependent effects on susceptibility to HSE have been described previously and it has been recognized in many viral infections of man and experimental animal species. Metabolic and hormonal changes, antibody responses, inhibitory substances, anatomical characteristics, and interferon production have all been suggested to explain this development of resistance [22], [23].

The virus in the DA susceptibility model penetrated the trigeminal nerve and the ipsilateral side of the brain stem after infection, subsequently spreading in contralateral and cranial direction within the CNS. While high virus titers were observed in the DA rats, no traces of HSV-1 could be detected in the resistant PVG, nor live virus was retrieved from the trigeminal ganglia or the brain stem using qRT-PCR [10] or immunohistochemistry [11]. These findings support the notion of an underlying genetic difference affecting the ability of HSV-1 to enter the nervous system of PVG rats.

Given the dichotomous difference in HSE susceptibility between the DA and PVG strains, HSV-1 infection of an F2 (DAxPVG.A) intercross was performed in order to identify the underlying genetic determinants in an unbiased fashion. The main finding of the linkage analysis was the identification of a new QTL on rat chromosome 4, *Hse1*.This QTL was the main regulator of disease both in males and females. In female rats the regulation of HSE seems to be more complicated as linkage analysis identified three additional smaller QTLs regulating different disease phenotypes suggesting the influence of other genes in disease development. The observed sex difference is in concordance with previous findings in the mouse, where sex dependent differences have been shown for the *Hrl* (Herpes resistance locus) locus and the *Sml* (sex modifier locus) locus which enhance resistance in females [8]. These two QTLs identified on mouse chromosome 6 correspond to a region towards the end of rat chromosome 4, outside the identified QTLs found in this study. However, *Hse1* was confirmed as the main disease regulating region by infecting congenic lines. The antigen-presenting lectin-like receptor gene complex (APLEC) located towards the end of chromosome 4 was previously reported to have a disease regulatory effect in a different model of HSV-1-induced encephalitis in the DA strain [15]. However, in this present study by infecting R17 congenic [24] rats with HSV-1, we could not confirm any HSE regulation by the APLEC genes region. The use of additional microsatellite markers, inclusion of more F2 rats with allelic recombinations and haplotype mapping in a set of inbred strains within the *Hse1* region made it possible to narrow down the confidence interval to a region of 3 genes. However, the finding that HSE resistant BN rats carry the same genotype as the DA rats in this region, suggests the existence of additional gene regions regulating HSE resistance in the BN rat. This demonstrates the significance of studying the genetic regulation in several inbred strains to enable the identification of all genes influencing the complexity of disease susceptibility.

Sequencing of the genes in the *Hse1* region identified a number of SNP variants mainly in the *Ccdc132*, *Calcr* and *Tfpi2* genes of the PVG.A strain, all of which were silent SNP variations. These silent variants do not change the amino acid sequence of the proteins; nevertheless these variants could possibly affect the translational stability, splicing or transcriptional control of these genes in PVG rats. Interestingly, we found that the mRNA expression of *Calcr* was significantly higher in the whiskers area both in the naïve and the infected PVG.A rats.

Two microRNAs (miRNAs) are present within the *Calcr*, rno-mir-489 and rno-mir-653. MiRNAs are short (22±3 nucleotides) RNA molecules that post-transcriptionally regulate gene expression by binding to 3′-untranslated regions (3′UTR) of target mRNAs, thereby inducing translational silencing and/or transcript degradation [25]. Both rno-miR-489 and rno-miR-653 are predicted to regulate a vast numbers of genes, making speculation on miRNA function difficult (http://www.microrna.org/microrna/home.do; http://www.targetscan.org/; http://www.mirdb.org/). Notably, there were no SNP variations in the sequence of mir-489 and mir-653 in DA and PVG. The levels of expression of the mature miR-489 and miR-653 were significantly higher at 5 dpi in PVG rats, resembling *Calcr* expression pattern in the whiskers area. However, both mature and mature* miR-489 and miR-653 were less abundant in the whiskers tissue, arguing against an influence on HSE susceptibility.

Calcitonin receptor (*Calcr*) is a seven-transmembrane G protein-coupled receptor which binds the peptide hormone calcitonin (32 amino acid residue), secreted by the parafollicular cells of the thyroid gland and is involved in the maintenance of the calcium homeostasis, particularly with respect to bone formation and metabolism. The ‘calcitonin family’ is a group of peptide hormones that share structural similarities with calcitonin and includes calcitonin gene-related peptide (CGRP), amylin, adrenomedullin and adrenomedullin 2 (intermedin). Heterodimerization of CalcR with any of the three receptor activity modifying proteins (RAMPs) forms the multimeric amylin receptors AMY1 (CT+RAMP1), AMY2 (CT+RAMP2), and AMY3 (CT+RAMP3) [26], [27]. The CalcR is expressed in a variety of tissues and cell types including the CNS, which also differs according to the developmental stage [28], [29]. In bone, it is restricted to osteoclasts, where it regulates their activity [30]. Little is known about the role of the CalcR in other tissues and it has not been previously implicated in infectious conditions.

Of great interest is the amylin hormone (also known as islet amyloid polypeptide (IAPP); 37 amino acid residues), which is similar in structure to calcitonin hormone and signals through *Calcr*. It is secreted by pancreatic β-cells parallel to insulin and is associated with type 2 diabetes development [31]. The *in vivo* use of CalcR agonist rat Amylin to modulate CalcR significantly enhanced the survival of DA rats after HSV-1 infection compared to controls. However, modulating an *in vitro* system using CalcR transfection of cell lines suggested that the presence of CalcR does not directly influence the infectivity of cells. Nevertheless, amylin signaling through CalcR could decrease the viral infection and/or replication inside cells through a more complex mode of action. In the same way the differences in expression levels together with the tissue localization of CalcR in DA and PVG rats could play a role in the degree of signaling through the receptor and thereby affect further viral spread. In addition, the high CalcR protein expression in the peri- and endoneurium layers in DA rats, together with the low *Calcr* expression in the trigeminal ganglia suggest a possible alternative route of axonal transport to the CNS causing encephalitis.

In conclusion, we here demonstrate that *Hse1* is the main genetic determinant for the susceptibility of DA rats to HSE and that it co-regulates differences in expression and tissue localization of *Calcr*. In addition, a direct clinical effect is evident by *in vivo* modulation of CalcR signaling. *In vitro* experiments, however, do not support a role of CalcR simply as a regulator of viral entry into cells, but rather to modulate infectivity and replication in a more complex fashion. Further studies are needed to define the contribution of the *Calcr* gene to HSE susceptibility, which may define novel mechanistic pathways involved in HSV-1 pathogenesis.

## Materials and Methods

### Ethics statement

This study was carried out in accordance with the guidelines from the Swedish National Board for Laboratory Animals and the European Community Council Directive (86/609/EEC) and approved by the Swedish ethical committee (Stockholm's North Ethical Committee - Stockholms Norra Djurförsöksetiska Nämd) (ethical permits N128/04, N340/08, N32/11).

### Rats

The inbred rat strains Dark Agouti-*RT1^av1^* (DA) and MHC- (RT1.AV1) congenic strain on Piebald Viral Glaxo-*RT1^av1^* background (PVG.A) were obtained from in-house breeding at the Animal Facility of Center for Molecular Medicine, Karolinska Institutet, Sweden.

All rats used in experiments were 45 days old when infected with 2×10^6^ PFU of neurovirulent HSV-1 (strain I-2762) in the whiskers area. Susceptible inbred strains debut with severe clinical HSE symptoms including coordination/balance disturbance, paralysis and/or die at 5 dpi. All rats were monitored for clinical HSE symptoms and weighed daily for 11 days, the set end time-point of the experiment. This was done considering that F2 animals possess different genetic composition compared to parental inbred strains and could present a different disease course. The HSE phenotypes followed in this study include; 1) Incidence: diseased rats were defined by the detection of clinical symptoms of HSE such as difficulties with coordination and balance, paralysis, weight loss >20% and death before day 10; whereas not diseased rats were defined as rats not showing any HSE symptoms. 2) Onset: defined by the first day of two consecutive days of weight loss or death. 3) Body weight change: measured by the differences in body weight at day 0, the start of the experiment and weight at 5 dpi. If animals showed body weight loss >20%, ataxia or paralysis the rats were euthanized and considered diseased.

#### F2 cohort for genome scans

The intercross between DA and PVG.A was generated by reciprocal breeding of parental rats, with F2 progeny originating from both DA and PVG.A female founders. A total of 239 F2 (DAxPVG.A) rats (120 females and 119 males) from in-house breeding and 30 additional F2 (DAxPVG.A) from a later in-house intercross were used in the study.

#### Congenic lines

The congenic lines DA.PVGc4-*Hse1* and DA.PVGc4-*Hse1*-R1were established from DA and MHC-identical PVG.A rats using a speed-congenic approach with marker assisted selection [32]. DA females were mated with male offspring selected from F2 (DAxPVG.A) with heterozygote alleles within chromosome 4 and *Hse1* QTL interval and against lower PVG.A background contamination using 86 microsatellite markers equally spaced throughout the genome (at 20 centimorgan cM intervals). DA females were used throughout the breeding program to ensure that mitochondrial DNA was inherited from the DA strain. A breeding pair selected from the N5 generation were crossed for two generations to produce homozygous DA.PVGc4-*Hse1* and DA.PVGc4-*Hse1*-R1 congenic rats, carrying PVG alleles between D4Kini3 at 27.81 Mb and D4Rat177 at 49.89 cM (114 Mb) and D4Mgh14 at 21.52 cM (36.5 Mb) respectively (Figure 3). Two females and 3 males from DA.PVGc4-*Hse1* as well as 3 females and 2 males from the DA.PVGc4-*Hse1*-R1 congenic rats were used for infection and phenotype observation. In addition, EAE congenics previously described [14] R2:DA.PVG (D4Rat23–D4Rat108), (12 females, 10 males); R11:DA.PVG (D4Rat103 – D4Mit12), (3 females, 2 males); R21:DA.PVG (OTO40.07 – D4Mit12), (8 females, 8 males) and APLEC congenic R17 [24] (D4Kiru12–D4Kiru55) (6 males) were used for infection and phenotype observation.

#### Haplotype mapping

Other strains were used for haplotype mapping, *i.e.* the Fisher 344 (F344: 5 females, 5 males) generously provided by Professor Holger Luthman, Lund University (Sweden); the spontaneously hypertensive rat (SHR: 5 females, 5 males) from Charles River Laboratories (Germany); the Fawn-Hooded (E3: 7 females, 5 males) generously provided by Professor Rikard Holmdahl, Lund University (Sweden); the Wistar Furth (WF: 5 females, 5 males) from Scanbur BK AB (Sweden); the Lewis (LEW: 3 females, 7 males); the August Copenhagen Irish (ACI: 10 males); the Brown Norway (BN: 5 females, 5 males) and the spontaneously type 1 diabetic Bio Breeding (BB: 1 female, 1 male) from in house breeding.

During the experiment, rats were kept in a full-barrier animal facility, at the Astrid Fagræus laboratory, within the Swedish Institute for Infectious Disease Control (SMI), in groups of 2 to 3 per cage under specific pathogen-free and climate-controlled conditions, with artificial 12 h light/dark cycles. The rats were housed in Eurotype IV polystyrene cages in enriched rat IVC (individually ventilated cage) system (Tecniplast, Italy) containing tin nests, aspen wood chips, aspen wood shavings and aspen chew blocks (Tapvei, Finland) and fed standard rodent chow (SDS, England) and water *ad libitum*. Ambient temperature was 21°C.

### Virus

HSV-1 virus strain I-2762 was used as described in our previous studies [10], [11]. After being thawed to room temperature, 100 µl virus suspension, containing 2×10^6^ PFU HSV-1 was injected instantaneously subcutaneously (*s.c.*) into the area of the whiskers' base unilaterally, on the right side, under 2% Isoflurane (Baxter) anesthesia.

### DNA isolation and genotyping

Genomic DNA was extracted from tail tips using a standard protocol [33]. Polymorphic microsatellite markers were selected from available Internet databases: Rat Genome Database (http://rgd.mcw.edu), Center for Genomic Research, Whitehead Institute/MIT (http://www-genome.wi.mit.edu/rat/public/), Ensembl (http://www.ensembl.org/) and The National Center for Biotechnology Information is available at (http://www.ncbi.nlm.nih.gov/). Oligo 6.0 software (National Bioscience) was used to design new microsatellite markers on rat chromosome 4 (D4Kini1–D4Kini16) from generated sequences available in Ensembl. Genotyping was performed using both fluorescent and radioactive methods. Flourophore-conjugated primers were purchased from Applied Biosystems (Carlsbad, CA, USA). PCR amplification was performed using a standard protocol and PCR products were separated using the electrophoresis capillary sequencer (ABI3730) and analyzed in the GeneMapper v3.7 software (Applied Biosystems). Radioactive PCR amplification was performed as previously described [34] with [γ-33P] ATP end-labeled forward primers (PROLIGO, France). The PCR products were size fractioned on 6% polyacrylamide gels and visualized by autoradiography. All genotypes were evaluated manually by two independent observers.

### Statistical and linkage analysis

Linkage analysis was performed using the statistical software R 2.8.0 (http://www.r-project.org) with the R/qtl package version 1.05–2 [35] and the marker map was obtained from Ensembl, version 45–2007. 180 individual rats from the F2 generation (total: 239, Table 1) were included in the linkage analysis, 74 females and 106 males. All rats were genotyped with 127 evenly spaced microsatellite markers providing 97% and 91% genome coverage with 25 cM to 20 cM spacing. Single-QTL genome scans were implemented by using the “Scanone” function of R/qtl with imputation method (step = 2.5, n.draws = 64) [36] for the following phenotypes: incidence, onset and body weight loss. The phenotype disease was also scanned using the binary model (step = 2.5) and similar results were obtained. The logarithm of odds (LOD) thresholds for a significant QTL [37] was obtained by performing permutations using 1000 simulations at the 95% and 63% confidence intervals (CI), respectively [38]. The threshold level of 95% was considered as significant linkage, whereas threshold level of 63% were considered as suggestive linkage at different given levels. Peak markers were in Hardy-Weinberg equilibrium. A confidence interval for linkage was defined by the utmost closest microsatellite marker after a LOD drop of 1.5 [39]. All traits were analyzed in the complete set including both males and females but also re-analyzed in each gender subgroup. To identify polygenic influence on HSE, we used forward selection to a model of 10 additive/interactive QTLs followed by backward elimination to the null model to identify a multiple-QTL model. The fit to a multiple-QTL model was used to statistically validate the independent effect of each identified QTL and percentage of phenotypic variance explained by identified multiple-QTL models. Allelic effects of QTLs identified in the multiple-QTL model and significance levels for phenotypic differences between parental strains were calculated using two-sided Student's t test using GraphPad Prism 5.0 (San Diego, CA, USA). A value of *P*≤0.05 was considered statistically significant.

### Sequencing

All sequences were generated from our genome-wide sequencing (Diana Ekman *et al*, manuscript in preparation). Genomic DNA from DA and PVG rats was used to construct 3 mate-pair libraries. The libraries were sequenced in the Uppsala Genome Center (Sweden) with SOLiD next generation sequencing version 2 and 3 machines. The sequences of the following genes: *F1LVX5_RAT*(ENSRNOG00000009135); *Fam133b* (ENSRNOG00000009163); *F1MA87_RAT*(ENSRNOG00000009258); *F1M477_RAT* (ENSRNOG00000039809); *F1LZE6_RAT* (ENSRNOG00000033874); *F1M482_RAT* (ENSRNOG00000026450); *D4A1X5_RAT* (ENSRNOG00000039801); *D4A1X6_RAT* (ENSRNOG00000039800; *D4A1X7_RAT* (ENSRNOG00000039799); *D4A1Y1_RAT* (ENSRNOG00000039798); *E9PTD6_RAT* (ENSRNOG00000009841); *Hepacam2* (ENSRNOG00000009711); *Ccdc132* (ENSRNOG00000009894); *Calcr* (ENSRNOG00000010053) and *Tfpi2* (ENSRNOG00000010513) were mapped to reference genome (BN) from Ensembl database release 66, with a >80% SNPs detection.

### Quantitative Real-Time PCR

#### mRNA genes expression

For the HSV-1 kinetic study of mRNA genes expression using qRT-PCR, 18 DA and 18 PVG.A rats were used (3 naïve, 5 controls, 5 rats taken at 2 dpi and 5 rats taken at 4 dpi from each strain). The procedure of tissue preparation from the whiskers area, trigeminal ganglia and brain stem and qRT-PCR was described in our previous study [11]. The following targets were analyzed: *Ccdc132*, *Calcr* and *Tfpi2*. Primer sequences for target genes and housekeeping genes are shown in Table S3. Statistical analysis was performed using the GraphPad Prism 5.05 program (San Diego, CA, USA). The significance levels of the differences between the DA and PVG.A strains over time were obtained by using two-way ANOVA with Bonferroni post-hoc test. A value of *P*≤0.05 was considered statistically significant. Primers used for qRT-PCR are listed in Table S3.

#### miRNA expression

To measure the expression of microRNA in the whiskers area using qRT-PCR, 9 DA (4 naïve and 5 taken at 5 dpi) and 10 PVG (5 naïve and 5 taken at 5 dpi) rats were used. Tissue homogenizing was performed in TRIzol using TissueLyser LT (Qiagen, Germany) and total RNA was isolated using standard TRIzol protocol (Invitrogen, Karlsruhe, Germany). RNA concentration and purity was determined by measurement of A260/A280 ratios with a NanoDrop ND-1000 Spectrophotometer (NanoDrop Technologies, Wilmington, DE, USA). RNA samples were immediately frozen and stored at −70°C.

Expression levels of selected miRNAs were confirmed by qRT-PCR analysis using TaqMan MicroRNA Assay Kit (Applied Biosystems, Barcelona, Spain) following the manufacturer's protocol. 10 ng RNA was used in each reaction. Specific assays were selected for both mature and mature* sequences of mir-489 (miRBase accession # MIMAT0003113 and MIMAT0017196 respectively), and mir-653 (miRBase accession # MIMAT0012838 and MIMAT0017361 respectively). miRNA expression levels were quantified by the BioRad CFX384 Real-Time Detection System and analyzed using CFX manager software v2.0 (Bio-Rad, Hercules, CA, USA). Relative quantification was measured using the 2−ΔΔCT method [40] and normalized against RNU6B for each sample. One-way ANOVA with Kruskal-Wallis test was performed using GraphPad Prism 5 (GraphPad Software, San Diego, CA, USA). A value of *P*≤0.05 was considered statistically significant.

### Immunohistochemistry

Twenty rats were used for immunohistochemistry, 5 DA and 5 PVG.A HSV-1 infected and dissected at 5 dpi, as well as 5 naïve DA and 5 naïve PVG.A. The procedure used and antibodies were described in a previous study [11]. In addition mouse anti-calcitonin receptor (1∶100) (Dako, Denmark) was used to stain expression of Calcitonin receptor in the different compartments.

### *In vivo* CalcR modulation experiment

Fifty-two male DA rats were used for the *in vivo* receptor modulating experiment. All rats were 44 days old when pre-treated 18 hours prior to HSV-1 infection. Under isoflurane anesthesia stimulation substances were injected unilaterally into the right whiskers' pad, where the HSV-1 was also injected the day after, under anesthesia. Sixteen rats were pre-treated with rat Amylin (0.05 mg/rat∼0.25 mg/kg body weight) (Bachem, Switzerland), 16 rats with Calcitonin (8–32) Salmon I (0.05 mg/rat∼0.25 mg/kg body weight) (Bachem, Switzerland), 10 rats with specially ordered scrambled peptide (0.05 mg/rat∼0.25 mg/kg body weight) (CASLO Laboratory, Lyngby, Denmark) consisting of the same amino acids as the rat Amylin, however in a reverse order and 10 rats were not pre-treated and used as infected controls. Rats were weighed and observed daily for disease symptoms until 11 dpi.

### Transfections and staining for flow cytometry

Transfections were performed with plasmids encoding for a myc-tagged version of the receptor activity-modifying protein 1 (RAMP1), necessary for modulating the CalcR signaling towards amylin, and with either the human CalcR or the rat CalcR (pcDNA3.1 and pcDNA1, respectively, all kind gifts from Professor Patrick Sexton, Monash University, Victoria, Australia). Briefly, HEK293T cells (human embryonic kidney cell line), which are semi-permissive to HSV-1 infection but do not express the CalcR, were plated in 24 wells plates at a density of 0,2×10^6^ cells/ml. Transfections were performed 24 hours later with 150 µg/well of all plasmids with Effectene transfection reagent (Qiagen) according to the manufacturer's instructions. Infections were performed 24 hours after transfection with the HSV-1 virus strain I-2762 at 3×10^5^ PFU/ml and 500 µl/well for 30 minutes at 37°C. Cells were subsequently washed once and rested for additionally 24 hours before staining. For the experiments with *in vitro* modulation of CalcR activity, the CalcR agonist rat Amylin (Bachem, Switzerland) was added either 4 hours prior to, and left during infection, being replenished after washing the infected cells; or was added only after infection. Experiments on the HSV-1 non-permissive rat mammary adeno-carcinoma cell line 13762 MAT B III (ATCC CRL-1666) were performed under the same conditions as described above.

Stainings for CalcR expression were performed on cells transfected for 24 hours. Briefly, cells were collected by pipetting and then fixed in Cytofix/cytoperm (BD Biosciences) for 20 minutes, washed and subsequently incubated with the Alexa Fluor 647 Conjugated mouse anti Myc-tag antibody (9B11) from Cell Signaling for the detection of RAMP1, and the rabbit polyclonal to CalcR (Ab11042) from Abcam followed by Alexa Fluor 488 Conjugated goat anti rabbit IgG (Molecular Probes) for the detection of both human and rat CalcR. For the assessment of virus infections, 24 hours infected cultures were collected as above, fixed and stained for RAMP1 as well as with the rabbit anti-HSV1 antibody B0114 (Dako) followed by an anti-rabbit secondary antibody as specified above. RAMP1 transfected cells (either alone or co-transfected with the human or rat CalcR plasmids) were gated and analyzed for the percentage of HSV-1 positive cells in the RAMP1-positive gate as well as for the mean fluorescence intensity (MFI) of the HSV-1 infected cell population. All samples were acquired by a Gallios flow cytometer and analyzed using Kaluza software (Beckam Coulter).

## Supporting Information

Table S1 **Linkage analysis for epistatic interaction identified additional sex specific QTLs influencing HSE.** Linkage analysis using forward selection with reverse elimination allowing for main and interactive QTLs identified QTLs on the following locations (in cM): Abbreviations: Var (%) = percent of phenotypic variance explained by the statistical model, *main QTL also identified in scanone.
(DOC)

Table S2 **Microsatellite markers designed for F2 (DAxPVG.A).** Microsatellite primers sequences designed around *Hse1* region on rat chromosome 4, the given names D4Kini- and the physical positions in Mb.
(DOC)

Table S3 **The qRT-PCR primers to candidate genes.** Primers sequences designed for candidate genes to measure mRNA expression level, as well as housekeeping gen.
(DOC)

## References

[1] Hjalmarsson A, Blomqvist P, Skoldenberg B (2007). Herpes simplex encephalitis in Sweden, 1990–2001: incidence, morbidity, and mortality.. Clin Infect Dis.

[2] Aurelius E, Forsgren M, Sköldenberg B, Strannegard O (1993). Persistent intrathecal immune activation in patients with herpes simplex encephalitis.. J Infect Dis.

[3] Sköldenberg B, Aurelius E, Hjalmarsson A, Sabri F, Forsgren M (2006). Incidence and pathogenesis of clinical relapse after herpes simplex encephalitis in adults.. J Neurol.

[4] Casrouge A, Zhang SY, Eidenschenk C, Jouanguy E, Puel A (2006). Herpes Simplex Virus Encephalitis in Human UNC-93B Deficiency.. Science.

[5] Zhang SY, Jouanguy E, Ugolini S, Smahi A, Elain G (2007). TLR3 deficiency in patients with herpes simplex encephalitis.. Science.

[6] Sancho-Shimizu V, Perez de Diego R, Lorenzo L, Halwani R, Alangari A (2011). Herpes simplex encephalitis in children with autosomal recessive and dominant TRIF deficiency.. J Clin Invest.

[7] Pereira RA, Scalzo A, Simmons A (2001). Cutting edge: a NK complex-linked locus governs acute versus latent herpes simplex virus infection of neurons.. J Immunol.

[8] Lundberg P, Welander P, Openshaw H, Nalbandian C, Edwards C (2003). A locus on mouse chromosome 6 that determines resistance to herpes simplex virus also influences reactivation, while an unlinked locus augments resistance of female mice.. J Virol.

[9] Lundberg P, Ramakrishna C, Brown J, Tyszka JM, Hamamura M (2008). The immune response to herpes simplex virus type 1 infection in susceptible mice is a major cause of central nervous system pathology resulting in fatal encephalitis.. J Virol.

[10] Bereczky-Veress B, Lidman O, Sabri F, Bednar I, Granath F (2008). Host strain-dependent difference in susceptibility in a rat model of herpes simplex type 1 encephalitis.. J Neurovirol.

[11] Bereczky-Veress B, Abdelmagid N, Piehl F, Bergstrom T, Olsson T (2010). Influence of Perineurial Cells and Toll-Like Receptors 2 and 9 on Herpes simplex Type 1 Entry to the Central Nervous System in Rat Encephalitis.. PLoS One.

[12] Lopez C (1980). Resistance to HSV-1 in the mouse is governed by two major, independently segregating, non-H-2 loci.. Immunogenetics.

[13] Kastrukoff LF, Lau AS, Puterman ML (1986). Genetics of natural resistance to herpes simplex virus type 1 latent infection of the peripheral nervous system in mice.. J Gen Virol.

[14] Marta M, Stridh P, Becanovic K, Gillett A, Ockinger J (2010). Multiple loci comprising immune-related genes regulate experimental neuroinflammation.. Genes Immun.

[15] Guo JP, Verdrengh M, Tarkowski A, Lange S, Jennische E (2009). The rat antigen-presenting lectin-like receptor complex influences innate immunity and development of infectious diseases.. Genes Immun.

[16] Hilton JM, Dowton M, Houssami S, Sexton PM (2000). Identification of key components in the irreversibility of salmon calcitonin binding to calcitonin receptors.. J Endocrinol.

[17] Malvy D, Ezzedine K, Lancon F, Halioua B, Rezvani A (2007). Epidemiology of orofacial herpes simplex virus infections in the general population in France: results of the HERPIMAX study.. J Eur Acad Dermatol Venereol.

[18] Karjala Z, Neal D, Rohrer J (2011). Association between HSV1 seropositivity and obesity: data from the National Health and Nutritional Examination Survey, 2007–2008.. PLoS One.

[19] Tunback P, Bergstrom T, Claesson BA, Carlsson RM, Lowhagen GB (2007). Early acquisition of herpes simplex virus type 1 antibodies in children–a longitudinal serological study.. J Clin Virol.

[20] Sancho-Shimizu V, Zhang SY, Abel L, Tardieu M, Rozenberg F (2007). Genetic susceptibility to herpes simplex virus 1 encephalitis in mice and humans.. Curr Opin Allergy Clin Immunol.

[21] Subramanian RP, Geraghty RJ (2007). Herpes simplex virus type 1 mediates fusion through a hemifusion intermediate by sequential activity of glycoproteins D, H, L, and B.. Proc Natl Acad Sci U S A.

[22] Johnson RT (1964). The pathogenesis of herpes virus encephalitis: II. A cellular basis for the development of resistance with age.. J Exp Med.

[23] Abel L, Plancoulaine S, Jouanguy E, Zhang SY, Mahfoufi N (2010). Age-Dependent Mendelian Predisposition to Herpes Simplex Virus Type 1 Encephalitis in Childhood.. J Pediatr.

[24] Lorentzen JC, Flornes L, Eklow C, Backdahl L, Ribbhammar U (2007). Association of arthritis with a gene complex encoding C-type lectin-like receptors.. Arthritis Rheum.

[25] Ambros V (2004). The functions of animal microRNAs.. Nature.

[26] Naot D, Cornish J (2008). The role of peptides and receptors of the calcitonin family in the regulation of bone metabolism.. Bone.

[27] Morfis M, Tilakaratne N, Furness SG, Christopoulos G, Werry TD (2008). Receptor activity-modifying proteins differentially modulate the G protein-coupling efficiency of amylin receptors.. Endocrinology.

[28] Pondel M (2000). Calcitonin and calcitonin receptors: bone and beyond.. Int J Exp Pathol.

[29] Mori I, Ishii A, Nakamura A, Nakamura M, Nakagomi N (2006). Expression and cellular localization of calcitonin receptor: RT-PCR and in situ hybridization studies.. Cell Mol Biol (Noisy-le-grand).

[30] Shen Z, Crotti TN, Flannery MR, Matsuzaki K, Goldring SR (2007). A novel promoter regulates calcitonin receptor gene expression in human osteoclasts.. Biochim Biophys Acta.

[31] Marzban L, Park K, Verchere CB (2003). Islet amyloid polypeptide and type 2 diabetes.. Exp Gerontol.

[32] Wakeland E, Morel L, Achey K, Yui M, Longmate J (1997). Speed congenics: a classic technique in the fast lane (relatively speaking).. Immunol Today.

[33] Laird PW, Zijderveld A, Linders K, Rudnicki MA, Jaenisch R (1991). Simplified mammalian DNA isolation procedure.. Nucleic Acids Res.

[34] Jacob HJ, Brown DM, Bunker RK, Daly MJ, Dzau VJ (1995). A genetic linkage map of the laboratory rat, Rattus norvegicus.. Nat Genet.

[35] Broman KW, Wu H, Sen S, Churchill GA (2003). R/qtl: QTL mapping in experimental crosses.. Bioinformatics.

[36] Sen S, Churchill GA (2001). A statistical framework for quantitative trait mapping.. Genetics.

[37] Lander E, Kruglyak L (1995). Genetic dissection of complex traits: guidelines for interpreting and reporting linkage results.. Nat Genet.

[38] Churchill GA, Doerge RW (1994). Empirical threshold values for quantitative trait mapping.. Genetics.

[39] Manichaikul A, Dupuis J, Sen S, Broman KW (2006). Poor performance of bootstrap confidence intervals for the location of a quantitative trait locus.. Genetics.

[40] Schmittgen TD, Livak KJ (2008). Analyzing real-time PCR data by the comparative C(T) method.. Nat Protoc.

