# Supplementary material for: The Calcitonin Receptor Gene Is a Candidate for Regulation of Susceptibility to Herpes simplex Type 1 Neuronal Infection Leading to Encephalitis in Rat
Source: PLoS Pathog. 2012 Jun 28;8(6):e1002753. doi: 10.1371/journal.ppat.1002753 (PMC3386237; doi:10.1371/journal.ppat.1002753)
Supplement: Table S1 — Linkage analysis for epistatic interaction identified additional sex specific QTLs influencing HSE. Linkage analysis using forward selection with reverse elimination allowing for main and interactive QTLs identified QTLs on the following locations (in cM): Abbreviations: Var (%) = percent of phenotypic variance explained by the statistical model, *main QTL also identified in scanone. (DOC) [file ppat.1002753.s001.doc]

**Supplementary Table 1 Linkage analysis for epistatic interaction identified additional sex specific QTLs influencing HSE.**

|  | FEMALES | | | | MALES | | | |
| --- | --- | --- | --- | --- | --- | --- | --- | --- |
|  | Var (%)  Total | Loci Chr (position) | Var (%)  in model | Significance level | Var (%) Total | Loci Chr (position) | Var (%)  in model | Significance level |
| Incidence | 92 | 4(35)*  10(35)  5(234)  10(139)  10(62)  10(139):10(62)  10(35):10(234) | 51  16  15  27  30  14  26 | <2e - 16  8.69e - 12  1.46e - 11  4.44e - 16  < 2e - 16  7.66e - 12 < 2e - 16 | 77 | 4(35)*  4(0)  17(48)  4(0):17(48) | 50  7  8  6 | 2e - 16  0.000376  0.000118  0.000196 |
| Onset | 99 | 4(35)*  8(24)  2(0)  6(132)*  16(143)  11(0)  4(35):8(24)  2(0):6(132)  16(143):11(0) | 54  42  26  24  8  5  32  21  5 | <2e - 16  <2e - 16  <2e - 16  <2e - 16  <2e - 16  <2e - 16  <2e - 16  <2e - 16  <2e - 16 | 97 | 4(35)* 6(12)  1(0)  1(29)  4(0)  14(26)  4(35):6(12)  1(0):1(29)  4(0):14(26) | 64  11  7  7  15  19  10  6  16 | <2e - 16  <2e - 16  <2e - 16  <2e - 16  <2e - 16  <2e - 16  <2e - 16  <2e - 16  <2e - 16 |
| WL d4 - d5 | 57 | 4(35)*  7(38)  4(35):7(38) | 49  22  18 | 2.34e - 09  0.000120  0.000101 | 89 | 4(35)  1(137)  4(0)  6(39)  14(0)  14(0)  4(35):1(137)  4(0):6(39)  14(26:14(0) | 33  14  7  8  7  7  10  7  5 | < 2e - 16 3.94e - 12 3.41e - 07 3.18e - 08 4.82e - 07 1.34e - 06 1.08e - 10 3.97e - 08 3.11e - 06 |
| WL d0 - d5 | 94 | 4(35)  3(133)  13(61)  3(133):13(61) | 35  37  36  36 | <2e - 16  <2e - 16  <2e - 16  <2e - 16 | 43 | 4(35)  9(30)  1(89)  4(0)  18(19)  3(61)  4(35):9(30)  1(89):4(0)  18(19):3(61) | 31  13  11  7  6  6  11  6  6 | < 2e - 16  2.61e - 09  5.62e - 08  2.23e - 05  1.31e - 05  5.67e - 05  2.90e - 09  6.42e - 06  2.19e - 05 |
| WL d0 - d3 | 45 | 10(98)  5(234)  10(98):5(234) | 43  6  5 | 7.86e - 07  0.000437  0.000336 | 20 | 3(32)  10(98) | 15  4 | 8.312638e - 05 |

Linkage analysis using forward selection with reverse elimination allowing for main and interactive QTLs identified QTLs on the following locations (in cM):

Abbreviations: Var (%) = percent of phenotypic variance explained by the statistical model, *main QTL also identified in scanone
