# Supplementary material for: The Calcitonin Receptor Gene Is a Candidate for Regulation of Susceptibility to Herpes simplex Type 1 Neuronal Infection Leading to Encephalitis in Rat
Source: PLoS Pathog. 2012 Jun 28;8(6):e1002753. doi: 10.1371/journal.ppat.1002753 (PMC3386237; doi:10.1371/journal.ppat.1002753)
Supplement: Table S2 — Microsatellite markers designed for F2 (DAxPVG.A). Microsatellite primers sequences designed around Hse1 region on rat chromosome 4, the given names D4Kini- and the physical positions in Mb. (DOC) [file ppat.1002753.s002.doc]

**Supplementary Table 2 Microsatellite markers designed for F2 (DAxPVG.A)**

| Microsatellite Marker | Mb position | Forward 5’ | Reverse 3’ |
| --- | --- | --- | --- |
| D4Kini14 | 21.80 | GGAATATGAGGATGAGAAAG | CCTGGTCTACAGAGTGAGTT |
| D4Kini15 | 22.00 | TGTGGAATACAGAGATTTCA | TGGATCTTCAAGTGTCAGTA |
| D4Kini16 | 23.52 | GGGACAGACATGACATTACT | GGCTAGGAAGATAACAGAAG |
| D4Kini1 | 24.38 | GTCTTGAGATTCCTTACCTTC | CTGTTAGGTGAAACAATGAAC |
| D4Kini2 | 27.28 | CCATTCTGGTTTATAGAGCT | ATCTCTACACTGAGCCATCT |
| D4Kini3 | 27.81 | ATTCTCCTGTCAGAAACTGT | TACAACTTCAGACATGTGGA |
| D4Kini4 | 28.30 | GCACAGTTGATAATCTACCA | ATGAACAGGAAGTCATGTGT |
| D4Kini5 | 28.37 | CTCTCCACCTCTTCTAATGT | CTAAACCCGTATATCAATGA |
| D4Kini6 | 28.45 | ACATGTGTGCATGTAGACAC | GATTGATGTTCACTCCACTC |
| D4Kini7 | 28.54 | TGACACACAGTGTAAGCTTT | AGAGAATAACCCTCTCCATA |
| D4Kini8 | 28.82 | ATGTCTTCCTCAGTGTGATC | TTAGAAAGGGAGGTATATCC |
| D4Kini9 | 28.84 | AATATCCACTTTCCCACTAC | GCTGGAGACAAAAGTTTTAC |
| D4Kini10 | 28.92 | AGAAAGGGAAAAACATATTC | CCTGATTTCACATCTATCCT |
| D4Kini11 | 28.93 | AAGTTTCTGGAACTTTGAAC | ATTTACTCTGGCTGCTACTC |
| D4Kini12 | 29.22 | TTCTTAATCCAGAGGTTTCT | GTTTCAAACACTGTGTTGAG |
| D4Kini13 | 30.40 | GGAAGTAGAAATACTTTGGG | ACCTTCTAAAGCCTTTAGAG |
