# Supplementary material for: The Calcitonin Receptor Gene Is a Candidate for Regulation of Susceptibility to Herpes simplex Type 1 Neuronal Infection Leading to Encephalitis in Rat
Source: PLoS Pathog. 2012 Jun 28;8(6):e1002753. doi: 10.1371/journal.ppat.1002753 (PMC3386237; doi:10.1371/journal.ppat.1002753)
Supplement: Table S3 — The qRT-PCR primers to candidate genes. Primers sequences designed for candidate genes to measure mRNA expression level, as well as housekeeping gen. (DOC) [file ppat.1002753.s003.doc]

**Supplementary Table 3 The qRT-PCR primers to candidate genes.**

| Primer | Forward 5’- 3’ | Reverse 5’- 3’ |
| --- | --- | --- |
| *Ccdc132* | CAGGACTATGACAGCGACAG | TTCCAACAAGCCTTATGACATTC |
| *Calcr* | CCAGAATGAAAAGGCGGAAC | GGAGTCAGTGAGATTGGTAGG |
| *Calcr.1b* | ACCTGGTGCGACGGGATC | GCAACTTATCACTTCACTGTC |
| *Tfpi2* | CAATGCCAACAATTTCCACAG | ACAAACCCAAGGAACTTTCTC |
| *Hprt* | CTCATGGACTGATTATGGACAGGAC | GCAGGTCAGCAAAGAACTTATAGCC |
| *Gapdh* | TCAACTACATGGTCTACATGTTCCAG | TCC CAT TCT CAG CCT TGA CTG |
